# Impact of sensory processing difficulties on academic performance and occupational balance in university students (PREstEO): Protocol for a cross-sectional and longitudinal study

**DOI:** 10.1371/journal.pone.0340983

**Published:** 2026-01-21

**Authors:** Daniel Prieto-Botella, Paula Peral-Gómez, Alicia Sánchez-Pérez, Berta Gándara-Gafo, Cátia Couço-Lucas, Paulo Fernandes, Sergio Serrada-Tejeda, Daniel Lloret, Paula Fernández-Pires

**Affiliations:** 1 Department of Pathology and Surgery, Miguel Hernández University, Alicante, Spain; 2 Being + Doing & Becoming (B+D&b) Occupational Research Group, Miguel Hernández University, Alicante, Spain; 3 Department of Pathology and Surgery, Miguel Hernández University, Alicante Institute for Health and Biomedical Research (ISABIAL), Alicante, Spain; 4 Department of Occupational Therapy, Health Integration and Promotion Research Unit (INTEGRA SAÚDE), Faculty of Health Sciences, University of A Coruña, La Coruña, Spain; 5 Center on Child Studies, University of Minho, Minto, Portugal; 6 Administração Regional de Saúde do Algarve, Faro, Portugal; 7 Department of Physical Therapy, Occupational Therapy, Rehabilitation and Physical Medicine, Rey Juan Carlos University, Madrid, Spain; 8 Department of Health Psychology, Miguel Hernandez University, Alicante, Spain; Hochschule Niederrhein - Campus Mönchengladbach: Hochschule Niederrhein - Campus Monchengladbach, GERMANY

## Abstract

**Background:**

Sensory processing difficulties (SPD) could significantly impact the daily functioning of university students, potentially affecting their academic performance and occupational balance (OB). However, this critical issue remains underexplored in the literature. The PREstEO study aims to investigate the association between SPD, OB, and academic performance among first-year university students and throughout their undergraduate studies. Additionally, the study will assess the prevalence of SPD and its associated factors in this population.

**Materials and methods:**

PREstEO is a longitudinal observational cohort study conducted at the Miguel Hernández University, Spain. The study aims to recruit first-year students (from September–October 2025) and follow them over four years (2025–2029). SPD will be assessed using the Adolescent/Adult Sensory Profile (AASP), while OB and academic performance will be measured using the Occupational Balance Questionnaire (OBQ-E) and students’ grade point average (GPA), respectively. Secondary measures include psychological distress, impulsivity, sleep quality, and problematic use of videogames, the internet, and psychoactive substances. Recruitment will involve in-person invitations and institutional media campaigns. Follow-ups will be conducted annually. Statistical analyses will include robust multiple linear regressions and generalized linear mixed models, adjusting for relevant sociodemographic and behavioral factors.

**Discussion:**

By adopting a longitudinal approach, PREstEO will provide novel insights into how sensory processing patterns interact with students’ daily lives over time. The findings will help to build strategies to enhance university students’ well-being and academic success.

## Introduction

In today’s world, we are constantly exposed to multiple sensory stimuli that influence how we interact with our environment [1,2]. Beyond the five traditional senses (sight, taste, smell, hearing and touch) that help us to perceive external sensory information, three additional senses provide essential insights regarding our own bodies. These senses are proprioception, which involves the sense of body position, force and self-movement; the vestibular sense, related to balance and coordination; and interoception, which provides information related to internal sensations such as thirst or hunger [2]. All these complex sensorial inputs are collected by specialized receptors and processed by the central nervous system to generate adaptative responses to different stimuli [3]. This dynamic process, known as sensory processing, is crucial for motor, social, emotional and cognitive development [4–6].

Dunn’s model of sensory processing explains that individuals respond to sensory input based on their neurological thresholds (low or high) and self-regulation strategies, resulting in four patterns: sensory sensitivity, sensory avoiding, sensory seeking, and low registration [7]. Sensory processing disorders or difficulties (SPD) occur when the brain cannot integrate sensory input effectively, including sensory reactivity issues such as hyperreactivity (as known as sensory over-responsivity, sensory sensitivity or sensory avoiding), hyporreactivity (as known as sensory under-responsivity or low registration), and sensory seeking (sensory craving) [8].

Current evidence suggests that SPD are highly prevalent in children with neurodevelopmental conditions [2], including autistic spectrum disorders [9,10], X fragile syndrome [11], alcohol fetal syndrome [12], attention-deficit hyperactivity disorder [13], and developmental coordination disorder [13]. However, SPD is not exclusive to these populations and can also affect children with typical development. Research indicates a SPD prevalence of 20–30% in typically developing children, with associated negative impacts on social participation, play, occupational performance in activities of daily living, learning, and academic performance [14,15].

Despite the potential impact of SPD on daily life, research on this topic in other populations, such as healthy adolescents and adults, is scarce and exploratory [16–18]. Most studies have focused on adults with specific medical conditions, creating a significant knowledge gap regarding the prevalence of SPD in healthy populations and its possible negative effects [19]. Additionally, the impact of SPD on university students has received limited attention, although the academic environment presents multiple sensory challenges, including noise, temperature fluctuations, and lighting conditions [18,20]. Processing these sensory inputs can be particularly difficult for university students with SPD, potentially affecting their concentration, attention, and performance during classes or exams [20]. Consequently, students with SPD may adopt maladaptive coping strategies to avoid sensory overloading situations, which could further affect their academic performance, social interactions, and overall participation in university life [20,21]. In fact, preliminary data suggests a potentially greater diversity of SPD among university students [20], which may adversely impact both their academic performance and occupational balance (OB).

OB is described as the subjective experience of having the right combination in quantity and variation of occupations in a particular occupational pattern [22]. Research conducted on the general adult population indicates that higher OB is associated with better subjective health, quality of life, life satisfaction, and reduced stress [23–26]. However, studies exploring OB among university students are limited. Preliminary findings suggest that higher OB in this population is related to higher mental and physical health, quality of life, and academic performance [27], but it can be negatively impacted by behaviors such as internet abuse [28]. Nevertheless, the potential influence of SPD on OB and academic performance in university students has not been deeply studied.

Therefore, the main objective of this study is to investigate the association between SPD, OB, and academic performance in first-year university students, as well as from the first to the fourth year of university. Additionally, our secondary objectives are:

1. To examine the prevalence of SPD and its associated factors in first-year university students.
2. To describe OB in first-year university students and identify its associated factors, as well as to explore changes between the first and fourth year of university.
3. To examine the association between problematic use of videogames, the internet, and psychoactive substances with OB and academic performance in first-year students and between the first and fourth year of university.
4. To evaluate the association between SPD and problematic use of videogames, the internet, and psychoactive substances in first-year students and from the first to the fourth year of university.

We hypothesize that, compared to university students without SPD, those with SPD will exhibit lower OB (Hypothesis 1) and poorer academic performance (Hypothesis 2) both during the first year of university and between the first and fourth year [20,21]. Additionally, we expect that students engaging in problematic behaviors, such as excessive videogames use or psychoactive substance consumption, will show lower levels of OB and academic performance (Hypothesis 3) [28]. Finally, we hypothesize that university students with SPD will have increased odds of engaging in problematic use of videogames, the internet, and psychoactive substances, both during the first year of university and between the first and fourth year (Hypothesis 4) [29–31].

## Materials and methods

### Study design and setting

The Sensory Processing, Academic Performance, and Occupational Balance in University Students (PREstEO) project is designed as a longitudinal, observational cohort study aiming to explore the association between SPD, OB, and academic performance among university students using quantitative methods. The research will be conducted at the Miguel Hernández University of Elche, a Spanish public university comprising four campuses with a student population of approximately 15000 students, including around 2600 first-year students. These new students will be evaluated at the beginning of their first semester (September-October 2025) and followed over four academic years/follow-ups, which corresponds to the typical duration of undergraduate studies in Spain (2025–2029).

This study protocol has followed the ObsQual guidelines for observational protocol reporting (S1 Appendix) [32], and the PREstEO resulting publications will follow the STROBE guidelines to ensure transparency reporting [33].

### Ethical considerations

This study protocol was approved by the Ethics Committee for Research Involving Medicines at the General University Hospital of Elche (PI 77/2024) and the Research Ethics and Integrity Committee of the Miguel Hernández University (DPC.PFP.240124). This study will be conducted in accordance with the Declaration of Helsinki, and all data will be managed in compliance with the Organic Law 3/2018 on the Protection of Personal Data and Guarantee of Digital Rights. Participation is voluntary, and university students will provide written informed consent before being enrolled. Data confidentiality will be maintained throughout the research process.

### Sample size estimation

Due to the limited research on SPD in healthy adults, an exact sample size calculation is not feasible. However, general population studies estimate that approximately 20% of individuals experience at least one difficulty in a sensory processing domain [34]. Based on this prevalence, a sample size of 820 first-year students (30% of the total incoming university students) would provide 80% power to estimate the prevalence with a ± 3% margin of error and an alpha criterion of 0.05. This calculation also accounts for a 20% attrition rate to ensure adequate sample retention. The resulting sample size will allow us to detect large effect sizes with statistical power greater than 80% in linear regression analyses, as recommended by Cohen [35].

### Participants

Target participants will be first-year bachelor’s degree students at the Miguel Hernández University of Elche. Recruitment will take place during the first two months of the academic year 2025–2026 (September–October), coinciding with the first semester. Invitations to participate will be extended in person following a brief presentation of the study, delivered before a lesson. Additionally, information about the project will be disseminated via institutional social media channels. Inclusion criteria include: a) being newly enrolled in a first-year bachelor’s degree program at Miguel Hernández University; b) being fluent in Spanish and c) providing written informed consent. Participants will be excluded if they meet any of the following conditions: a) reporting a neurological condition affecting sensory processing (e.g., brain injury, sleep disorders, neurodegenerative diseases); b) reporting developmental disabilities (e.g., autism spectrum disorder, Asperger syndrome, attention-deficit/hyperactivity disorder) or c) having an active mental health disorder diagnosed by a healthcare professional.

### Procedure

**Fig 1:**
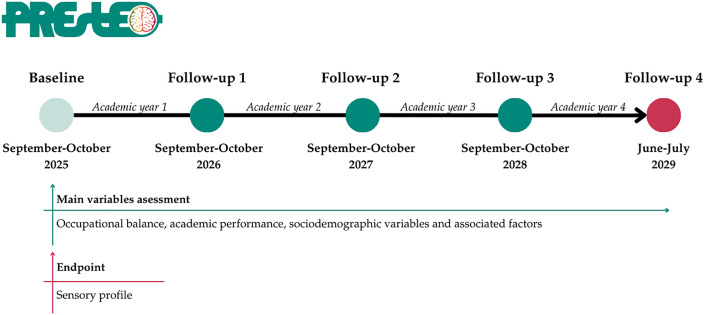
Study scheme of the PREstEO cohort. Enrollment of first-year students will occur between September and October 2025. At this initial stage, their sensory profile will be assessed. PREstEO is a longitudinal study that begins with baseline recruitment in the first year of university and includes follow-ups at the beginning of each academic year, continuing until the end of the fourth year in June-July 2029.

The study scheme summarizing the PREstEO project is presented in Fig 1.

a) Enrollment and first data collection

Before the start of the 2025–2026 academic year, vice deans from the different bachelor’s degree programs (n = 28) will be contacted via email and phone to invite them to participate in this study. Upon receiving their consent, professors will be contacted to request a few minutes before or after their classes to present the study and collect data. The objective of these contacts is to secure in-person sessions for participant enrollment. Additionally, at the beginning of the 2025–2026 academic year, a dissemination campaign providing information about PREstEO will be launched through institutional media channels.

Evaluators will attend the scheduled sessions, present the study to students, and invite their participation. Students who agree to enroll will sign a written informed consent form and self-report the 15-minute assessment. At this initial stage, data will be collected on SPD, OB, academic performance (as used for university admission), sociodemographic information, and associated factors.

b) Follow-ups

Students’ institutional emails will be collected at each assessment for follow-up purposes and linked to a numeric participant code in a separate database. This database will be accessible only to the principal investigator, who will not be involved in statistical analysis. The final database will contain only numeric codes, ensuring that no personally identifiable information is included.

During the fourth academic year, the follow-ups will be performed following the above-mentioned in-person process. Data collection will include OB, academic performance, sociodemographic information and associated factors. SPD information will only be collected at the initial time point, while academic performance will represent the previous academic years. For instance, the 2026 follow-up (Fig 1) will assess academic performance from the first academic year, and this pattern will continue accordingly. For this reason, to collect academic performance for the fourth academic year, the final follow-up will take place in June–July 2029.

c) Sample retention interventions

Participant dropouts are a common challenge in longitudinal studies [36]. To enhance sample retention, a dedicated campaign will be implemented throughout the project. This campaign will include an annual giveaway, where participants’ chances of winning will increase based on the number of completed assessments, incentivizing continued participation. Additionally, a social media campaign will be conducted targeting participants for community-building and to provide follow-up reminders with informative videos and study updates [37].

### Measures

A summary of all measures that will be collected in this study at each follow-up is presented in Table 1.

**Table 1:** Summary of the data collection plan for the PREstEO project.

Outcomes	B	F1	F2	F3	F4	Assessment tool
**Main outcomes**						
Sensory processing difficulties	X					AASP
Occupational balance	X	X	X	X	X	OBQ
Academic performance	X	X	X	X	X	Ad hoc
**Secondary outcomes**						
Stress	X	X	X	X	X	DASS-21
Depression	X	X	X	X	X	DASS-21
Anxiety	X	X	X	X	X	DASS-21
Impulsivity	X	X	X	X	X	Plutchnik
Sleep	X	X	X	X	X	ISI
Videogames addiction	X	X	X	X	X	GASA
Internet addiction	X	X	X	X	X	IAT
Cannabis, tobacco and alcohol use	X	X	X	X	X	Ad hoc
Subjective physical activity	X	X	X	X	X	Ad hoc
Adherence to Mediterranean diet	X	X	X	X	X	MEDAS
**Sociodemographic information**						
Age	X					Ad hoc
Sex	X					Ad hoc
Ethnicity	X					Ad hoc
Weight and height	X	X	X	X	X	Ad hoc
Current bachelor’s degree	X	X	X	X	X	Ad hoc
Educational level	X					Ad hoc
Marital status	X	X	X	X	X	Ad hoc
Parental status	X	X	X	X	X	Ad hoc
Work status	X	X	X	X	X	Ad hoc
Socioeconomic status	X	X	X	X	X	Ad hoc
Residency during the academic year	X	X	X	X	X	Ad hoc
Preterm birth	X					Ad hoc

Abbreviations: B, Baseline; F, Follow-up; AASP, Adolescent/Adult Sensory Profile; OBQ, Occupational Balance Questionnaire; DASS, Anxiety, depression and stress scale; ISI, Insomnia Severity Index; GASA, Game Addiction Scale for Adolescents; IAT, Internet Addiction Test; MEDAS, Mediterranean Diet Adherence Screener.

a) Main variables

**Sensory processing:** The Spanish version of the Adolescent/Adult Sensory Profile (AASP) will be used to identify SPD [38]. It consists of 60 items divided into six sensory processing factors (taste/smell, movement, visual, tactile, activity level, and auditory processing) and four quadrants that reflect sensory patterns (sensation seeking, sensory sensitivity, sensation avoiding, and low registration). Each item’s score ranges from 5 (almost always, 95% of the times) to 1 (hardly ever, 5% of the times). Reference values for the Spanish AASP are also available [39].

To establish typical reference values within the population and differentiate typical sensory processing patterns from altered ones, a statistical criterion based on the normal distribution was applied to each quadrant and factor [39]. Mean values and standard deviations (SD) were calculated, allowing for classification into the following categories: significantly lower than most (scores ≥2 SD below the mean for individuals of the same age), lower than most (between 1 and 2 SD below the mean), similar to most (scores between −1 and +1 SD), higher than most (between 1 and 2 SD above the mean), and significantly higher than most (≥2 SD above the mean). Intraclass correlation coefficients (ICC) indicated strong reliability, with values exceeding 0.9 for each quadrant. Cronbach’s alpha ranged from 0.69 to 0.73 across quadrants, demonstrating acceptable internal consistency [39].

**Occupational Balance:** OB will be assessed using the Spanish version of The Occupational Balance Questionnaire (OBQ-E) [40]. The OBQ-E evaluates occupational performance based on satisfaction with the quantity and diversity of occupations. It consists of 13 items, scoring from 0 (completely disagree) to 5 (completely agree). The total score ranges from 0 to 65 points, with higher scores indicating better OB. The OBQ-E showed good internal consistency (Cronbach-α = 0.87), intraclass reliability (ICC = 0.87), and test-retest reliability (rho = 0.83) [40].

**Academic Performance:** The grade point average (GPA) calculated as the sum of all bachelor’s degree grades divided by the total number of credits, will be used to assess academic performance at university. In the Spanish educational system, grades range from 1 to 10, with a minimum of 5 required to pass. At each follow-up, the GPA corresponding to the previous academic year/s will be collected. This GPA will be available for all participants on the university website.

At baseline, participants will report their GPA used for university admission. This average refers to the Spanish university entrance examination. This score is calculated as the average of the final grades from the last two years of high school combined with the total score from the examination, with a maximum of 14 points. For students who completed a vocational training program, the secondary school GPA is replaced by the average grade from the two years of vocational training. To ensure comparability, all university admission scores will be standardized using z-score normalization.

b) Secondary variables

**Anxiety, depression and stress:** The Spanish version of the Depression, Anxiety, and Stress Scale (DASS-21) will be used to assess these variables [41,42]. This instrument measures psychological distress through 21 items, divided into three subscales (depression, anxiety, and stress) each comprising seven items. Participants assess how much each statement applied to them over the past week using a scale from 0 (not at all) to 3 (very much or most of the time). The DASS-21 has demonstrated excellent internal consistency, with a Cronbach’s alpha of 0.90 [41,42].

**Impulsivity:** It will be assessed using Plutchik’s impulsivity scale, a 15-item questionnaire scored on a four-point scale (0 = never, 3 = almost always). The Spanish-validated version of this tool presented a strong internal consistency (Cronbach-α = 0.90), and good test-retest reliability (rho = 0.89) [43].

**Sleep:** The Spanish version of the Insomnia Severity Index (ISI) will be used to evaluate the subjective severity of insomnia and its impact on quality of life [44]. The ISI consists of five items, each rated on a five-point scale (0 = no problem, 4 = severe problem), with higher scores indicating a greater negative impact. Higher scores indicate a higher negative impact. This version showed good internal consistency (Cronbach-α = 0.82) [44].

**Videogames addiction:** This outcome will be measured using the Spanish version of the Game Addiction Scale for Adolescents (GASA) – Short form [45]. The scale comprises seven items, evaluating seven criteria (salience, tolerance, mood modification, relapse withdrawal, conflicts, and problems). Among Spanish university students, this version presented good internal consistency (Cronbach-α = 0.83) [45].

**Internet addiction:** The Spanish version of the Internet Addiction Test (IAT) will be used to identify problematic Internet use [46]. This test consists of 20 items scored using a five-point scale (0 = never, 5 = always). This 20-item assessment is scored on a five-point scale (0 = never, 5 = always). Based on the total score, users are classified into two groups: (1) normal users or those without problems (<40 points) and (2) problematic internet users (≥40 points). Among Spanish university students, this version presented excellent internal consistency (Cronbach-α = 0.91) and high test-retest reliability (rho = 0.89) [46].

**Cannabis, tobacco, and alcohol use:** The frequency, quantity, and onset of cannabis, tobacco, and alcohol consumption will be assessed using the questions from the 2023 XIV edition of the Survey on Drug Use in Secondary Education in Spain (ESTUDES) [47].

**Subjective physical activity:** Physical activity will be assessed using a self-reported question where individuals rate their perceived activity level on a 6-point scale, ranging from “very sedentary” to “very active”.

**Adherence to the Mediterranean diet:** It will be measured using The Mediterranean Diet Adherence Screener (MEDAS), a validated Spanish questionnaire consisting of 14 items, each scored either 0 or 1, resulting in a total score ranging from 0 to 14. The first 12 items assess the frequency of food consumption, while the last two focus on preferred cooking fats and types of meat consumed. A score above 9 reflects strong adherence to the Mediterranean diet [48].

c) Sociodemographic data

At baseline, sociodemographic information regarding age (years), sex (male, female), ethnicity (as proposed by the European Union Agency for Fundamental Rights), educational level (other university studies or vocational training) and preterm birth (yes, no) will be collected. Additionally, the following data will be assessed both at baseline and at the different follow-ups: weight (kg), height (cm), current bachelor’s degree program, marital status (single, married/living common-law, divorced/separated, widowed), parental status (children number), work status (employee, self-employed, unemployed, time off work/sick leave), socioeconomic status (low, middle, high), and residency during the academic years (own house, family house, students house/university residency).

### Statistical analysis

The statistical analysis will be performed using the latest available version of R software (R Foundation for Statistical Computing, Vienna, Austria; http://www.R-project.org). All tests will be bilateral, with a significance level set at 0.05. The normality of quantitative variables will be assessed using the Kolmogorov–Smirnov test with Lilliefors correction.

Students’ sociodemographic characteristics will be analyzed using frequencies and percentages for qualitative variables. For quantitative variables, the mean and standard deviation or the median and interquartile range will be reported, depending on the normality of the data distribution. Differences between groups will be assessed using the chi-square test or Fisher’s exact test for qualitative variables, and either Student’s t-test/Mann–Whitney U-test or ANOVA/Kruskal–Wallis test for quantitative variables. The analysis will explore: (1) students with SPD (scores in any quadrant classified as lower/higher or significantly lower/higher than most) versus those with typical sensory processing (scores in all quadrants classified as similar to most) and (2) students with lower and significantly lower scores across any quadrant versus those with higher and significantly higher scores in any quadrant versus those with typical sensory processing. Additionally, individual quadrant scores and sensory processing levels will be described to address the first part of secondary objective 1.

At baseline, we will conduct several cross-sectional statistical analyses. Firstly, we will examine the association between SPD (exposure variable), OB, and academic performance in first-year university students (main objective) using robust multiple linear regression models from the robustbase R package [49]. Models will be adjusted for sociodemographic and secondary variables with a p-value <0.2 in the bivariate analysis [50]. Additionally, variables that alter the main effects by more than 10% after a backward–forward elimination procedure will also be included [50]. The Variance Inflation Factor (VIF) will be calculated using the car package in R to assess multicollinearity in the regression models [51]. Robust multiple linear regression models will also be applied to analyze objectives 2, 3, and 4 in the first-year assessment. Furthermore, associated factors (exposure variables) related to SPD in the first year will be evaluated using multiple Poisson regression models [52,53]. To obtain prevalence ratios, we will estimate robust variance using the Huber sandwich method [52,53].

Before conducting the longitudinal analysis at follow-up 4, we will follow the procedure suggested by Hair et al. to address missing data [54]. First, we will analyze the types and percentages of missing data to ensure they do not exceed the recommended thresholds. Second, we will use Little’s test from the naniar R package to assess whether data are missing completely at random (MCAR) [55]. Finally, we will perform a sensitivity analysis to evaluate the impact of missing data on the study results by comparing the main outcomes between completers and imputed values. Missing data will be handled using multiple imputation via chained equations [56].

The longitudinal analysis will be conducted using generalized linear mixed models with the lme4 R package [57], and p-values for the model outputs will be obtained using the lmerTest package [58]. Variance across participants and university campuses will be modelled as random effects, while group (as previously defined) and time (follow-up points) will be treated as fixed effects. The group × time interaction will be examined to assess the trajectories of each group over time. Covariates will be accounted for using the same approach as in the cross-sectional analysis. To estimate the magnitude of between-group differences at baseline and the 4-year follow-up (and between follow-ups), effect sizes (Cohen’s d) will be calculated. Effect sizes will be interpreted according to Cohen’s conventions: 0.20 as small, 0.50 as medium, and ≥0.80 as large [35].

## Discussion

PREstEO is designed as a longitudinal observational study with the novel objective of exploring the impact of SPD on OB and academic performance among university students at Miguel Hernández University in Spain. Additionally, the study aims to determine the prevalence of SPD, and the factors associated with these variables in this population. The recruitment of first-year students will take place between September and October 2025, with follow-up assessments conducted at the start of each academic year, continuing until the completion of the fourth year in June–July 2029.

To date, no previous longitudinal scientific evidence has specifically focused on describing SPD, OB, and academic performance in university students. Moreover, the factors potentially associated with SPD and OB (both considered health-related outcomes) [19,23–25] have yet to be identified in this population. Therefore, our project, by exploring and analyzing the associations between all these variables, represents a pioneering effort at a global level. The findings could provide valuable insights for developing policies, strategies, and institutional actions specifically tailored to university students, particularly given the complex and demanding nature of the university environment [20].

Nevertheless, our study presents several limitations and strengths that must be acknowledged. First, the success of this study relies on the proper recruitment and follow-up management of participants, as dropouts could significantly impact the project. Given our goal to recruit nearly 30% of first-year university students, we have outlined several measures to address potential low participation. Specifically, 1) we will strengthen the recruitment campaign by expanding dissemination channels, including email outreach, and increasing the frequency of giveaways; 2) we will establish direct contact with student organizations and groups to enhance promotion; and 3) we will collaborate with the University of Alicante and Rey Juan Carlos University to extend recruitment efforts. Despite these strategies, we will initiate the recruitment process using a standardized questionnaire, provide weekly participation updates, and maintain communication with professors to support recruitment efforts [36,37]. Additionally, we have set a target sample size 20% higher than the average dropout rate in Spanish universities (10–15% in in-person bachelor’s degrees) to mitigate potential attrition [59].

Despite these efforts, the generalizability of our findings may be limited. Research volunteers are often less diverse than the general population, and socioeconomically disadvantaged students may find it harder to retain in longitudinal cohort studies [60]. Moreover, given that our sample consists exclusively of university students, the results may not be directly applicable to other populations. However, we believe that the PREstEO project has the potential to generate novel and applicable knowledge that will significantly contribute to understanding SPD in university students. By exploring these challenges, our study will provide a foundation for improving OB and academic performance in this specific population, ultimately informing institutional policies and support strategies developed to their needs.

Another key strength of this project is the use of standardized, adapted, and validated assessment tools to evaluate both primary and secondary outcomes. In particular, the use of the AASP will provide essential and novel insights into SPD in university students. Implementing these validated instruments will enhance the reliability of measurements and strengthen the overall evaluation protocol, ensuring robust and replicable findings. Although all these measures are self-reported and may be subject to recall and self-report biases, each instrument has demonstrated strong psychometric properties, ensuring reliable and valid data collection. To mitigate potential biases, we will implement standardized administration procedures and emphasize clear instructions for participants. Additionally, the longitudinal design of the study will allow us to track trends over time, reducing the impact of individual variability and enhancing the robustness of our findings.

Finally, this study will contribute to expanding the scientific evidence on SPD in university students, offering valuable insights into factors that can be targeted to improve mental health, OB, and academic performance in this population. The findings of the PREstEO project could contribute to developing targeted occupational therapy interventions aimed at enhancing students’ OB, and academic performance, such as creating sensory-friendly study environments, tailored coping strategies, and personalized academic accommodations. All findings generated from this research will be disseminated to both the educational and scientific communities, to develop informed policies and encourage new research avenues to further explore these critical issues.

## Supporting information

S1 Appendix Observational study protocol recommended items (ObsQual) checklist. (DOCX)
