# Supplementary material for: Impact of sensory processing difficulties on academic performance and occupational balance in university students (PREstEO): Protocol for a cross-sectional and longitudinal study
Source: PLoS One. 2026 Jan 21;21(1):e0340983. doi: 10.1371/journal.pone.0340983 (PMC12822976; doi:10.1371/journal.pone.0340983)
Supplement: S1 Appendix — (DOCX) [file pone.0340983.s001.docx]

| **Appendix 1. ObsQual Checklist: Observational study protocol recommended items.** | | | |
| --- | --- | --- | --- |
| **Item** | **Item no.** | **Explanation** | **Page number in the protocol** |
| Title | 1 | Indicate the study’s design with a commonly used term in the title. | 1 |
| Introduction Background/rationale | 2 | Explain the scientific background and rationale for the investigation being reported. This section requires references from current literature. | 2,3 |
|  | 3 | The study is necessary in comparison with current evidence. It is important to state the importance of this study in the absence of evidence. If evidence is already available to address the study objectives, consider other objectives of study that addresses the knowledge gap. | 2-4 |
| Objectives | 4 | State specific objectives, including any prespecified hypotheses. An objective should be SMART (Specific, Measurable, Achievable, Realistic, Time-bound). | 3,4 |
| Methods and Study design | 5 | Present key elements of study design early in the paper. A one paragraph of overview of the study design. | 4 |
| Setting | 6 | Describe the setting, locations, and relevant dates, including periods of recruitment, exposure, follow-up, and data collection. This section may be an independent heading or as a second paragraph in the method section. Some information might have already been described in the first paragraph (overview of study design). Describe the details as if the readers can replicate the study. | 4,6, Figure 1 |
| Participants | 7 | Cohort study—Give the eligibility criteria, and the sources and methods of selection of participants. Describe methods of follow-up Eligibility/selection criteria are designed to address inclusion or exclusion of participants with certain characteristics (factors) that may influence the analysis. | 5,6 |
|  | 8 | Case-control study—Give the eligibility criteria, and the sources and methods of case ascertainment and control selection. Give the rationale for the choice of cases and controls. | Not applicable |
|  | 9 | Cross-sectional study—Give the eligibility criteria, and the sources and methods of selection of participants Same as above Item 7 explanation on eligibility criteria in cohort study section. | 5,6 |
|  | 10 | Cohort study—For matched studies, give matching criteria and number of exposed and unexposed | Not applicable |
|  | 11 | Case-control study—For matched studies, give matching criteria and the number of controls per case Same as above Item 10. | Not applicable |
| Variables | 12 | Clearly define all outcomes, exposures, predictors, potential confounders, and effect modifiers. Give diagnostic criteria, if applicable | 7-9, Table 1 |
| Data sources/management | 13 | For each variable of interest, give sources of data and details of methods of assessment (measurement). Describe comparability of assessment methods if there is more than one group. | 7-9 |
| Bias | 14 | Describe any efforts to address potential sources of bias. Provide rationale of certain methods used to address potential bias. | 6,10-12 |
| Study size | 15 | Explain how the study size was arrived at. | 4 |
| Quantitative variables | 16 | Explain how quantitative variables will be handled in the analyses. If applicable, describe which groupings will be chosen and why. | 10 |
| Statistical methods | 17 | Describe all statistical methods, including those used to control for confounding. The methods such as T-test, Chi-square, regression, etc. must be stated. | 10,11 |
|  | 18 | Describe any methods used to examine subgroups and interactions. | 10,11 |
|  | 19 | Explain how missing data will be addressed. | 10 |
|  | 20 | Cohort study—If applicable, explain how loss to follow-up will be addressed. | 10-12 |
|  | 21 | Case-control study—If applicable, explain how matching of cases and controls will be addressed. | Not applicable |
|  | 22 | Cross-sectional study—If applicable, describe analytical methods taking account of sampling strategy. | 10,11 |
|  | 23 | Describe any sensitivity analyses. | 10 |
| Appendices/Biological specimens | 24 | Plans for collection, laboratory evaluation, and storage of biological specimens for genetic or molecular analysis in the current trial and for future use in ancillary studies, if applicable | Not applicable |
